# Potential financial loss and risk factors for hamstring muscle injuries in elite male Brazilian soccer players: a season-long prospective cohort pilot study

**DOI:** 10.3389/fspor.2024.1360452

**Published:** 2024-09-24

**Authors:** Otaviano Oliveira-Júnior, Tim J. Gabbett, Natalia F. N. Bittencourt, Roberto C. Quintão, Guilherme F. Reis, João G. Claudino, Rodrigo C. P. Lasmar, Amanda A. O. Leopoldino

**Affiliations:** ^1^ Post Graduate Program in Health Sciences, Faculdade Ciências Médicas de Minas Gerais, Belo Horizonte, Brazil; ^2^ Medical Department of Professional Soccer, Clube Atlético Mineiro, Belo Horizonte, Brazil; ^3^ Gabbett Performance Solutions, Brisbane, QLD, Australia; ^4^ Health Innovation and Transformation Centre, Federation University, Ballarat, VIC, Australia; ^5^ Soccer Science Center, Federal University of Minas Gerais (UFMG), Belo Horizonte, Minas Gerais, Brazil; ^6^ Sports Science, School of Applied Sciences (FCA), University of Campinas (Unicamp), Limeira, São Paulo, Brazil

**Keywords:** muscle strain, eccentric knee flexor strength, isokinetic, football (soccer), global positioning satellite system (GPS)

## Abstract

**Purpose:**

The aim of this pilot study was to analyze the potential financial loss and a range of potential risk factors for hamstring muscle injuries in elite Brazilian soccer.

**Methods:**

Thirty-four male players (age: 25 ± 6 years; stature: 180 ± 8 cm; body mass: 78 ± 9 kg; minutes played in matches: 2243 ± 1423 min) from an elite professional soccer club were monitored during a 12-month season. Muscle injury was identified by magnetic resonance imaging and the severity was defined according to the number of days away: minimal (1–3 days), mild (4–7 days), moderate (8–28 days), severe (>28 days). Potential financial loss due to the team's under achievements was determined. Dorsiflexion range of motion, eccentric knee flexor strength and isokinetic tests were performed during the pre-season. Association between dependent variables and the occurrence of injury was evaluated.

**Results:**

Nine hamstring muscle injuries with moderate severity were found in 8 athletes. Recovery time was 22 days off the field on average. Potential financial loss was $-43.2 million USD and earnings on merit money was 21%. Previous injury, increased flexor deficit 60° /sec and increased flexor fatigue index 300°/sec were all associated with a greater chance of hamstring muscle injury. Ankle dorsiflexion range of motion was significantly lower in the injured group (35.6 ± 3° vs. 39.1 ± 4.9°; *p* = 0.017, effect size = −0.74).

**Conclusion:**

High financial burden was found in elite Brazilian soccer during one full season. Injured athletes had high hamstring fatigue index, knee flexor strength deficit, ankle range of motion restriction and previous hamstring muscle injury when compared to non-injured athletes. Therefore, preventive approaches in professional soccer players with previous hamstring injuries should be a priority.

## 1 Introduction

Soccer played in the strongest national leagues in the world (1) requires increasingly high levels of physical fitness and intensive training of the athletes (2–4). Specifically, muscle strength is essential in sports with high physical demands, such as soccer (5, 6). Soccer is characterized by sequences of high-intensity actions: running, jumping, and explosive technical skills that require acceleration and deceleration (7, 8). In this context, well-developed physical qualities, such as joint mobility, muscle strength and flexibility, and the ability to perform high-speed running are essential in preparing high-performance teams (5, 9–11). Due to the intense match demands, training loads of elite soccer players are also high (12, 13), explaining around 76% of the common variance between matches and training sessions (14), which may explain, at least in part, the high incidence of muscle injuries. Ekstrand et al. (2) investigated players from the UEFA Elite Club for 21 seasons between 2001/02 and 2021/22 and observed that hamstring injuries represented 19% of all reported injuries, with the proportion of all injuries increasing from 12% during the first season to 24% in the last season. Hamstring injuries are also reported to be one of the injuries most likely to negatively impact team performance in the UEFA Champions League or Europa League (15).

Although it is frequently reported on social networks and sports channels, in the scientific literature the few studies found report that the costs involved in hamstring muscle injuries are very high (16) and the negative impacts of injuries related to team performance are responsible for most of the financial loss for soccer clubs (17). For example, an English Premier League team lost approximately ₤45 million per season due to injury–related decrements in performance (i.e., 80% <=> the team's under achievement due to injured athletes + 20% <=> salaries paid to injured athletes) (17). Due to the negative impact of hamstring muscle injuries on costs and loss of finances, different strategies have been used in order to prevent these injuries. Amateur soccer players who used the FIFA 11 Program for injury prevention incurred fewer hamstring muscle injuries and fewer severe injuries, reducing the mean cost of hamstring muscle injuries for players who performed the program (18). Researchers have also analyzed modifiable and non-modifiable risk factors^1^ for hamstring muscle injuries (19–33).

Regarding non-modifiable risk factors, athletes with a history of hamstring muscle injury had a three times greater risk of suffering a new injury in the same muscle group (25). Additionally, the risk increased fivefold when this injury occurred in the same season (25). The literature also describes the relationship of previous anterior cruciate ligament (ACL) injuries with hamstring muscle injuries (25). It is thought that a reduction in knee neuromuscular control may contribute to hamstring injuries post ACL rupture, and that these changes may persist for up to three years following ligament reconstruction, especially with the use of the hamstring surgical graft (19, 28).

Although there are considerably more modifiable risk factors that may influence hamstring injury risk, there is conflicting findings in the scientific literature (25, 33). Variables related to hamstring muscle strength have traditionally been measured using isokinetic dynamometer equipment (23, 29, 32, 33) and eccentrically using the NordBord Hamstring Testing® (and similar) devices (21, 22, 27, 31). Despite fatigue being described as one of the predisposing factors for muscle injuries (26), there are limited investigations within the literature (20). The range of motion of the ankle and knee have been investigated as risk factors for hamstring muscle injuries, although older age and a history of hamstring muscle injury are the strongest risk factors (25). Passive hamstring and ankle dorsiflexion ranges of motion have been described as weak risk factors for hamstring injury (33). Nevertheless, decreased ankle mobility may predispose the hamstring muscles to a greater risk of strain injury, presumably due to an alteration in the biomechanical position of the foot (34), leading to a reduction in horizontal strength during sprinting (35), generating an increase in hamstring work (33). In addition to the physical characteristics of athletes, the increased high-speed running demand may also increase injury risk, although exposure to these higher loads is necessary to develop the physical qualities to protect against injury and produce better physical and technical performance (36–41). In a recent study of elite soccer players, players who completed moderate volumes of high-speed running (701–750 m: OR: 0.12, 90%CI: 0.08–0.94) and sprint running (201–350 m: OR: 0.54, 90%CI: 0.41–0.85) were at reduced injury risk compared to those who completed low volumes of high-speed running (≤674 m) and sprinting (≤165 m) (10).

In summary, there is conflicting evidence regarding the risk factors for hamstring muscle injuries, and there is a high incidence of hamstring injuries and associated financial burden, particularly in soccer players from the strongest national leagues in the World. Therefore, this season-long prospective cohort pilot study aimed to verify the risk factors for hamstring muscle injuries assessed via isokinetic testing, weight bearing lunge testing, eccentric hamstring strength and external workload monitoring and potential financial loss in elite Brazilian soccer.

## 2 Methods

### 2.1 Study design and ethical aspects

This study used a prospective cohort experimental design. We studied a professional soccer team in the first division of the Brazilian Championship. This study was approved by the Research Ethics Committee of the Faculdade Ciências Médicas de Minas Gerais (CAAE: 15737819.6.0000.5134), according to the rules of Resolution 196/96 of the National Health Council regarding research involving human beings. All athletes manually signed the informed consent form. Consent to use the medical records was obtained by signing the club's Letter of Consent.

### 2.2 Sample

The study was carried out in an elite Brazilian soccer club. Sample size was calculated in order to estimate hamstring muscle injury prevalence in soccer players. The equation used is described below (42):

$$n=p\;(1-p)\;{\left(\frac{Z_{1-\;\alpha /2}\;+\;Z_{1-\;\beta }\;}{d}\right)}^{2}$$

with *p* as prevalence estimated from Ekstrand et al. (3), $Z_{1-\;\alpha /2}\;$ and $Z_{1-\;\beta }$ as percentiles of the standard normal distribution associated with the significance and power of the test, respectively, and *d* as minimum difference to be tested. Thus, considering the significance level of 5%, minimum power of 80% to test a minimum difference of 25% in relation to the prevalence of injury of 37% (3), at least 30 athletes were needed in the sample. Four more athletes were included to cover possible sample losses (42). All athletes on the squad were recruited and remained throughout the process, except for 5 athletes who left the club during the season. They were male, aged between 18 and 39 years, and participated in official games for 12 consecutive months in the 2019 season. Goalkeepers were excluded because of their unique physical demands relative to field players, and consequently, did not have the same risk of exposure to hamstring injuries. Athletes who left the club for any reason and were unable to undergo testing were excluded.

### 2.3 Procedures

Data were collected for 12 months in the 2019 season by checking medical records (season start) and applying a questionnaire to athletes containing physical parameters and past medical history. Aspects of the athlete's profile such as: age, body mass index, position, previous injuries in the last two years (muscle—hamstring muscle injury, ankle inversion sprain) and in the last three years (ACL injury in the knee) (19, 25), distance covered at high-intensity running speed monitored by GPS, congested game schedule that may be related to the physical quality and the efficient execution of game actions were verified. After collecting the sociodemographic and clinical data, a complete assessment of the lower limbs was performed to characterize their functional profile. The British classification was used to assess injury severity based on clinical examination and magnetic resonance imaging (43).

Hamstring muscle injury was defined as the micro or macro rupture of fibers of the tendon muscle complex of the biceps femoris, semitendinosus and semimembranosus, either by an eccentric mechanism during running or by excessive stretching of the thigh, including both the first episode and any recurrences which limited the athlete to participate in training or games (4). In addition, the *Fédération Internationale de Football Association* (FIFA) criteria were used to assess the severity of injuries according to the number of days of absence: minimal (1–3 days), mild (4–7 days), moderate (8–28 days), severe (>28 days) (44). Based on the Croisier (23), recurrent hamstring injury was identified when more than one occurrence during the season.

Potential financial loss due to the team's under achievements was determined by summing the merit money deficit and salaries paid to injured athletes. The merit money is based solely on each club's final position in the competition (17). Thus, the merit money deficit is the difference of the team position and the champion team in United States Dollar (USD). Merit money paid in Brazilian Real (BRL) was converted in USD using the yearly average exchange rate, i.e., USD 1 = BRL 3.946 (45). There were 4 competitions (i.e., Brazilian Championship Serie A, Brazil Cup, CONMEBOL Libertadores, CONEMBOL Sudamericana) that paid merit money for this club in the season and all these values are in the public domain. Due to the institution's confidentiality agreements, salaries paid to injured athletes were taken from the soccer club's financial statements which is also a public file. For the calculation, the total amount of salaries paid to athletes with image rights was used. First, the salary paid per athlete per day (i.e., salary paid/athlete/day) was calculated, and then this salary paid daily was multiplied by the total of off-field days (i.e., recovery time).

### 2.4 Instruments and variables

#### 2.4.1 Isokinetic evaluation

The isokinetic strength assessment was performed using a Biodex System Pro 4® isokinetic dynamometer (Biodex, NY, USA). First, a brief familiarization and ten-minute warm-up was performed on an ergometric bicycle (46). According to the protocol, testing was performed for each knee with an angular velocity of 60° /s and 300° /s with concentric knee flexion and extension movement under intense verbal command from the examiner, with an interval of 90 s between each test. Five and 30 repetitions were used, respectively, for each velocity. Flexor and extensor peak torque, flexor fatigue index (i.e., measured by the equipment protocol that considers the fatigue index as the percentage of strength loss between the first and the last third of the 30 repetitions performed at a speed of 300° /s), and hamstring/quadriceps ratio variables were used [(47); intraclass correlation coefficient = 0.87 by (48)]. The interval time between legs was around 15 min.

#### 2.4.2 Weight bearing lunge test (WBLT)

**Figure 1:**
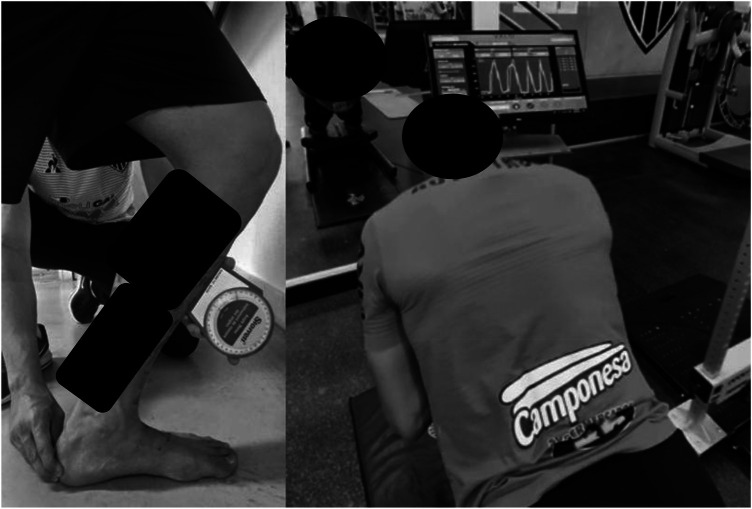
**(A)** placement of the lunge test. Source: Author's personal archive. **(B)** NordBord Hamstring Testing® placement. Source: Author's personal archive. *The black boxes were necessary to make this image unidentifiable.

The Weight Bearing Lunge Test (WBLT) was performed to assess ankle dorsiflexion limitation. The WBLT is a validated test with good reliability (49, 50). The test was performed with the athlete in an orthostatic position. In this position, participants performed an anterior advancement movement of the unilateral tibia over the talus in maximum dorsiflexion until the detachment moment of the calcaneus from the ground (Figure 1A). The tibia angulation was measured with an inclinometer using the AM-2 model from Starrett. The averages of the 3 measurements for each ankle dorsiflexion range of motion were used in further analyses.

#### 2.4.3 Nordbord hamstring testing

The hamstring eccentric muscle strength test was performed using the NordBord Hamstring Testing® device which has previously been validated, with normative data for biceps femoris injury (27). To perform the test, the participants remained in a kneeling position on a padded plate with their ankles secured to the lateral malleolus by individual “shin guards”, which were fixed on commercially available uniaxial load cells (MLP-1K; Transducer Techniques, Inc., Temecula, CA). They were asked to perform one set of three maximal “Nordic exercises with hamstrings” bilaterally (Figure 1B) to obtain the following variables: relative peak force_highest_ (highest/body mass), between-limb imbalance (highest), relative peak force_average_ (average/body mass), between-limb imbalance (average) (22).

#### 2.4.4 External workload monitoring

Global positioning system (GPS) devices (model SPI HPU; Gpsports Systems, Australia) were used to collect external load in training and games. The system uses data interpolation from 5 Hz GPS units and 100 Hz accelerometers to provide displacement and velocity data at 15 Hz. Each player wore a device (with a weight of 76 g and dimensions of 48 × 20 × 87 mm), placed in the thoracic region. The device was turned on 15 min before the activity and turned off immediately after the session. Data were recorded and transferred to a computer, then analyzed in the equipment's software program (Team AMS R1 2016).

External workload comparison of athletes with and without hamstring muscle injury was performed using data from 28 days before the athlete's injury (51). In addition, comparisons were made with another athlete from the same position and similar physical demands, who had not sustained injury. We used the variable absolute high-speed running (HSR) distance covered above 19.8 km/h for each athlete in different time frames to analyze the difference in acute and chronic load between the groups with and without injury. The four weeks of training and game data before the injuries were sustained were compared with athletes of the same position and similar demand. We used 3 different acute and chronic loading windows: the first-time window was 7/28 days, as it is the most commonly used period in the literature (51). The time frames of 4/16 days and 1/4 days were performed to capture the training demand closest to the injury. In these analyses, all participation time from training sessions and matches (regardless of the time played) were included. Counts were performed during the 4, 16, and 28 day periods before the day the injury occurred. Absolute HSR load performed in these periods were used in the analyses.

### 2.5 Statistical analysis

Qualitative variables were presented as frequencies, and quantitative variables as mean ± standard deviation (median). Quantitative variables were submitted to the Shapiro–Wilk normality test and if necessary, the corresponding non-parametric test was applied.

The association between the variables and injury occurrence was evaluated by a simple binary logistic model, and the results presented as an odds ratio (OR) and respective 95% confidence intervals (CI). Variables with *p* < 0.20 in the association analysis were included in a multiple binary logistic model, which reached the final model using the backward strategy. The significant variables and age independent of significance remained to control effects. Quality of fit analysis was performed using the Hosmer–Lemes how test. Edges's g was used to analyze the effect size (52) and the following classifications were used to measure the magnitude of ES: ES (Large effect >0.80; moderate effect 0.20–0.80; small effect <0.20) (53). The analysis was performed using the R version 4.0.2 program and *p* < 0.05 was considered significant.

## 3 Results

The sample consisted of 34 soccer players, of which 8 (24%) sustained 9 hamstring muscle injuries. The distribution of the injury by position was: attackers = 4 (44.4%), midfield = 3 (33.3%), full-back = 1 (11.1%) and defender = 1 (11.1%). This single recurrence of the hamstring injury took 47 days to recover (i.e., severe). A total of 202 off-field days were needed for recovery time and an average of 22 days (with median of 18 days) to recovery per hamstring muscle injury. Thus, the hamstring injuries were classified as moderate severity. The average age of the athletes was 25 ± 6 years and the average Body Mass Index (BMI) was 24.07 ± 1.10 kg/m^2^. Thirteen players were attackers (38.2%), nine were midfield (26.5%), six were full-backs (17.6%) and six were defenders (17.6%). Previous hamstring injury (observed in 19 limbs, 27.9%), previous ACL surgery (observed in 6 limbs, 8.8%) and ankle sprain (observed in 22 limbs, 32.4%) were associated with a greater risk of hamstring injuries. Higher BMI was associated with a reduced injury risk (OR 0.14, 95% CI 0.02; 0.50, *p* = 0.014) and previous hamstring muscle injury was associated with an increased injury risk (OR 7.08, 95% CI 1.64; 37.36, *p* = 0.011) (Table 1).

**Table 1:** Characterization of lower limbs according to the occurrence of hamstring injury.

Characteristics	No injury *(n = 59)*	Injury *(n = 9)*	OR (95%CI OR)	*P*-value	ES
Age (year)	25.1 ± 5.7	26.2 ± 6.0	1.03 (0.91; 1.16)	0.593	0.18
BMI (kg/m^2^)	24.2 ± 1.1	23.1 ± 0.4	0.14 (0.02; 0.50)	**0**.**014**	**−1.06**
Position					
Attacker	22 (37.3%)	4 (44.4%)	–	–	–
Sideback/Winger	11 (18.6%)	1 (11.1%)	0.50 (0.02; 3.91)	0.556	
Midfield	15 (25.4%)	3 (33.3%)	1.10 (0.19; 5.70)	0.909	
Defender	11 (18.6%)	1 (11.1%)	0.50 (0.02; 3.91)	0.556	
Previous HMI	13 (22%)	6 (66.7%)	7.08 (1.64; 37.36)	**0**.**011**	–
Previous ACL surgery	5 (8.5%)	1 (11.1%)	1.35 (0.07; 9.94)	0.796	–
Ankle sprain	18 (30.5%)	4 (44.4%)	1.82 (0.41; 7.69)	0.410	–

HMI, hamstring muscle injury, *n*, number; BMI, body mass index; ACL, anterior cruciate ligament; ES, effect size; yr, years; kg, kilogram; m^2^, square meter.

Values in bold highlight results with statistical significance.

In the evaluation of the balance/strength variables (Table 2), higher knee-flexor fatigue index at 300°/sec was associated with an increased risk of injury (OR 1.15, 95% CI 1.04; 1.31, *p* = 0.013), while increased dorsiflexion range of motion decreased the risk of injury (OR 0.80, 95% CI 0.62; 0.97, *p* = 0.040). There was no difference in external workload of injured athletes paired with non-injured athletes (Table 3). Furthermore, the same non-significant result was found for the minutes played in matches between groups (injured athletes: median = 1,426 [1,116–1,787] minutes vs. non-injured: median = 1,651 [678–3,309]; *p*-value = 0.39532).

**Table 2:** Characterization of balance/strength variables according to the occurrence of hamstring injury.

Characteristics	No injury *(n = 59)*	Injury *(n = 9)*	OR (95%CI OR)	*P*-value	ES
Peak torque extensors 60° /sec (*N*)	352.2 ± 45.8 (355.5)	364.5 ± 52.4 (372.1)	1.01 (0.99; 1.02)	0.459	0.26
Extensor deficit 60° /sec (%)	8.3 ± 8.1 (4.4)	7.3 ± 5.4 (7.2)	0.98 (0.88; 1.07)	0.725	−0.13
Peak torque flexors 60° /sec (*N*)	193 ± 28.4 (193.6)	200.8 ± 27.3 (194.5)	1.01 (0.98; 1.04)	0.438	0.28
Flexor deficit 60° /sec (%)	8.6 ± 7.3 (5.7)	13.1 ± 9.8 (10.8)	1.07 (0.98; 1.17)	0.112	0.59
Extensor work 60° /sec (w)	336.2 ± 47.6 (338.3)	335 ± 56.2 (353.9)	0.99 (0.98; 1.01)	0.941	−0.02
Extensor work deficit 60° /sec (%)	11.2 ± 8.3 (8.9)	11.6 ± 6.9 (9.6)	1.01 (0.92; 1.09)	0.880	0.05
Flexor work 60° /sec (w)	213.7 ± 33.4 (207.4)	214 ± 39.5 (203.9)	1.00 (0.98; 1.02)	0.979	0.01
Flexor work deficit 60° /sec (%)	11.9 ± 6.7 (11.3)	15.7 ± 7.3 (14.2)	1.08 (0.98; 1.20)	0.128	0.56
Agonist/Antagonist ratio 60° /sec (%)	55.5 ± 7.9 (54.1)	55.6 ± 7.2 (57.3)	1.00 (0.91; 1.10)	0.947	0.01
Flexor fatigue index 300° /sec (%)	49.5 ± 8.9 (51.7)	58.1 ± 8.5 (56.6)	1.15 (1.04; 1.31)	**0**.**013**	**0**.**97**
Relative peak force_highest_ (highest/body mass; N/kg)	5.2 ± 0.8 (5.2)	5.2 ± 0.9 (4.8)	0.96 (0.36; 2.43)	0.927	0.00
Between-limb imbalance (highest;%)	7.9 ± 5.9 (8)	6.4 ± 3.5 (5)	0.95 (0.81; 1.08)	0.472	−0.26
Relative peak force_average_ (average/body mass; N/kg)	5.0 ± 0.6 (4.9)	4.8 ± 0.8 (4.6)	0.70 (0.21; 2.11)	0.540	−0.32
Between-limb imbalance (average; %)	7.4 ± 5.8 (7)	6.1 ± 4.3 (6)	0.95 (0.81; 1.08)	0.515	−0.23
Ankle dosiflexion ROM (°)	39.1 ± 4.9 (38)	35.6 ± 3.0 (35)	0.80 (0.62; 0.97)	**0**.**040**	**−0**.**74**

median; HMI, hamstring muscle injury; ROM, range of motion; AVG, average; *n*, number; sec, seconds; ES, effect size. ^W^Wilcoxon Mann–Whitney test, ^T^Student's *t*-test for independent samples; N, Newton; %, percentage; w, watts; kg, kilogram; °, degrees.

Values in bold highlight results with statistical significance.

**Table 3:** Comparison of the performance of athletes with and without hamstring injuries in paired GPS assessment.

Characteristics	Mirror athletes *(n = 9)*	Injured athletes *(n = 9)*	OR (95%CI OR)	*P*-value	ES
Absolute HSR in 90 min game (m)	644 ± 163 (617)	694 ± 182 (730)	1.00 (0.99; 1.01)	0.525	0.29
A/C 7/28D	0.92 ± 0.14 (0.92)	0.98 ± 0.39 (0.89)	2.14 (0.07; 87.10)	0.657	0.20
*7-day workload* (m)	144 ± 37 (132)	128 ± 69 (118)			
*28-day workload* (m)	156 ± 24 (159)	137 ± 61 (123)			
A/C 4/16D	1.03 ± 0.49 (1.20)	1.51 ± 0.87 (1.20)	3.46 (0.78; 39.65)	0.198	0.68
*4-day workload* (m)	159 ± 95 (139)	171 ± 105 (135)			
*16-day workload* (m)	155 ± 34 (168)	129 ± 61 (157)			
A/C 1/4D	1.38 ± 1.34 (2.10)	1.60 ± 0.70 (1.90)	1.24 (0.49; 3.32)	0.643	0.21
*1-day workload* (m)	290 ± 339 (113)	228 ± 242 (210)			
*4-day workload* (m)	159 ± 95 (139)	171 ± 105 (135)			

median; HMI, hamstring muscle injury; GPS, global position system; HSR, high-speed running, distance traveled at high speed, >19.8 km/h); A, acute); C, chronic); D, days; *n*, number; ES, effect size); m, meters.

Previous hamstring injury (*p* = 0.032), greater knee-flexor deficit 60° /sec (*p* = 0.005) and greater knee-flexor fatigue index 300° /sec (*p* = 0.009) were associated with a greater risk of hamstring muscle injury (Table 4).

**Table 4:** Factors associated with hamstring injury.

Characteristics	OR	95%CI OR	*P*-value
Constant	4.636×10^−13^	(2.673×10^−25^; 2.716×10^−06^)	**0**.**008**
Age	1.094	(0.849; 1.393)	0.454
Previous HMI	13.254	(1.611; 23.705)	**0**.**032**
Flexor deficit 60° /sec	1.301	(1.113; 1.625)	**0**.**005**
Flexor fatigue index 300° /sec	1.443	(1.173; 2.122)	**0**.**009**

HMI, hamstring muscle injury; OR, odds ratio. Hosmer–Lemes how *P*-value test 0.198.

Values in bold highlight results with statistical significance.

**Figure 2:**
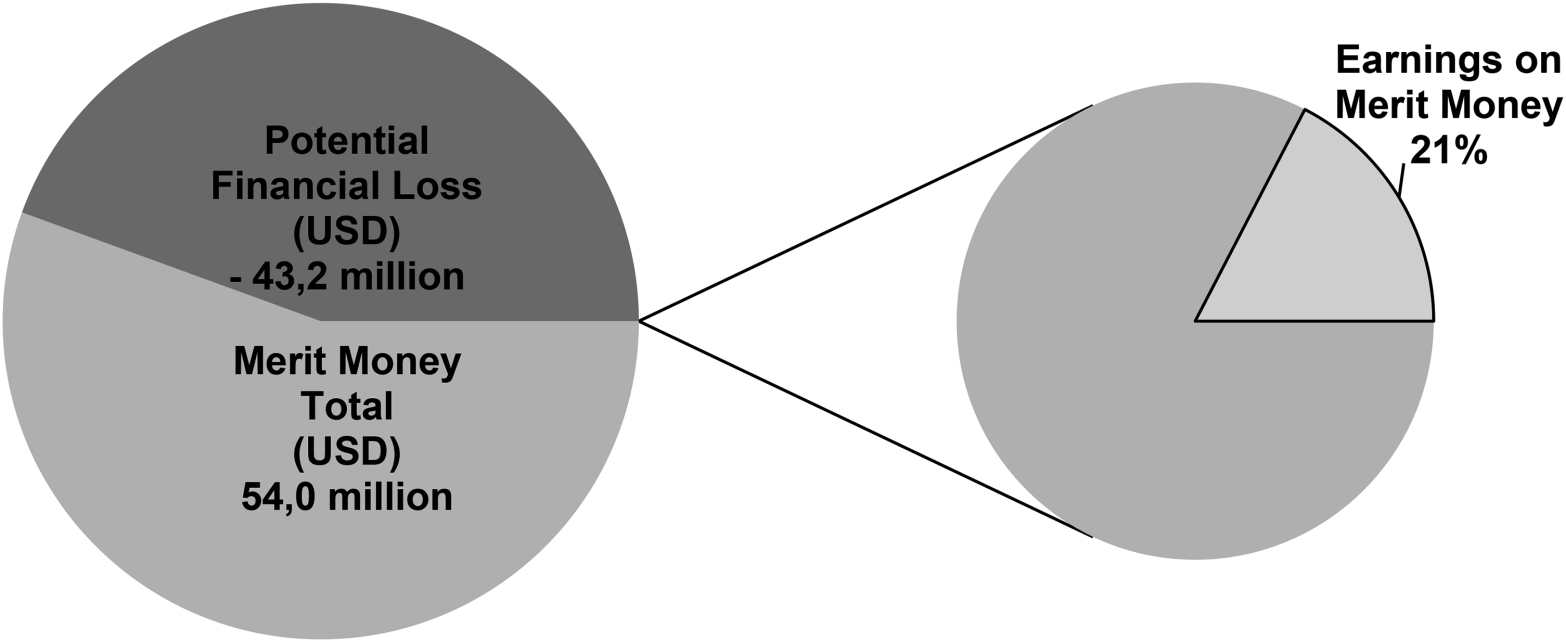
Potential financial loss and earnings on merit money.

Potential financial loss was approximately $ −43.2 million USD (98.7% <=> the team's under achievement due to injured athletes + 1.3% <=> salaries paid to injured athletes) and 21% of the merit money that was obtained by the soccer team (Figure 2; Supplementary Table S1).

## 4 Discussion

This pilot study aim is verifying the risk factors for hamstring muscle injuries assessed via isokinetic testing, weight bearing lunge testing, eccentric hamstring strength and external workload monitoring and potential financial loss in elite Brazilian soccer. Thus, the present pilot study showed an association between hamstring muscle injury and a higher rate of fatigue in the hamstrings, increased knee-flexor deficit at 60° /sec, dorsiflexion range of motion limitation, and previous hamstring muscle injury. It is noteworthy that there was no difference in the high-speed running variables and other isokinetic muscle variables or eccentric hamstring strength, nor previous ACL injuries, ankle sprains, and age. Thus, resistance to hamstring fatigue, knee flexor asymmetry between limbs and poor ankle mobility should be a focus of interventions in elite soccer players in Brazil, since these factors can increase the risk of hamstring muscle injury. Furthermore, the team's under achievement due to injured athletes plus salaries paid to injured athletes resulted in $ −43.2 million USD of potential financial loss and 21% receipt of merit money.

In agreement with several studies, a higher proportion of injured players had previous hamstring muscle injuries (54, 55). In a meta-analysis of 19 studies, Green et al. (25) demonstrated that previous hamstring muscle injury increased the risk of a new hamstring injury by 3 times (RR = 2.7; *p* < 0.001) whereas in the present study it was 7 times. Although the present study had a small sample size, 67% of the injured group had a previous hamstring muscle injury while only 22% of the uninjured group had a previous hamstring injury (*p* = 0.011).

Contrary to the results presented in the literature on isokinetic muscle performance (56), the data from the present study showed no difference between groups for traditional variables such as hamstrings and quadriceps concentric and eccentric torque. However, the present study showed differences between groups for hamstring fatigue index. The injured group had higher fatigue index at 300° /s compared to the non-injured group (58.1 ± 8.5 vs. 49.5 ± 8.9; ES = 0.97). Some studies demonstrate that fatigue could lead to changes in neuromuscular coordination patterns and potentially increase the risk for hamstring muscle injuries (26, 57). Fatigued muscles have a lower capacity to produce force and are more prone to injury from eccentric contractions compared to non-fatigued muscles (26). Regarding eccentric strength variables measured from the NordBord®, the current results corroborate the evidence from the last meta-analysis (25) that there is no association with hamstring muscle injuries. The NordBord® test was performed with a shortened hamstrings position, and in contrast, the most common mechanism of hamstring muscle injuries occurs in the elongated position during a sprint (3, 25). In addition, for the effective use of this device, there is still a need for consensus to identify the best procedures, definitions, and calculations for evaluation of the eccentric strength of hamstrings in different sports settings (22).

Literature exploring the association of ankle dorsiflexion range of motion and hamstring injury risk are conflicting (25, 58). In agreement with the meta-analysis by Green et al. (25), we found no association with the risk of hamstring muscle injury. However, there was a significant difference in this variable between groups in the present study. The mean range of motion in the injured group was 35.6 ± 3°; while the mean in the group without injury was 39.1 ± 4.9° (ES = −0.74; *p* = 0.017). The difference in range of motion between groups was above the measurement error reported in the literature (i.e., 1.3°–2.8°), reinforcing that the values were clinically relevant (58). A smaller ankle dorsiflexion range of motion can limit ipsilateral limb take-off during running, and therefore, require high force production by the hamstrings to perform the running task at high speed. Thus, this continuous overload can increase the mechanical demand on this muscle group and increase injury risk, meaning that the pre-season assessment that detects restricted ankle range of motion could possibly be used in practice as a screening tool to prevent hamstring muscle injuries. However, further studies are needed to confirm this hypothesis.

Due to the high physical demands of soccer and the congested schedule with short breaks between games, many studies have investigated the association of training load and games played with muscle injuries (46, 59). Some authors have shown that exposure to inappropriate load can increase injury risk up to 5 times (RR = 5.1), with a greater number of accelerations performed over a three-week period associated with greater injury risk in young soccer players (36). However, other studies have shown little association between training load (59) or the congested calendar (60) and injuries. The results of the present study showed that there was no difference between groups for training and game external load. Differences in findings between the present and previous studies may reflect differences between the Brazilian and European calendar, with Brazilian players competing in more games across a season (46, 61). The incidence of injury has previously been compared between South American and European teams. Findings suggest no differences between competitions, reinforcing the hypothesis that although match congestion is higher, injury risk is not increased in South American clubs (62).

According to a recent meta-analysis on the topic, some isolated risk factors were strongly correlated with hamstring muscle injury (25). Considering the multifactorial nature of the etiology of musculoskeletal injuries, investigating the interactions of factors has a crucial role in the occurrence of this injury (63). Despite recognizing the complex nature of muscle injuries, this study did not perform complex and non-linear analyses due to the sample size, as nine injuries were analyzed in just one soccer team and a single season. However, it is the first study to analyze the difference between groups using strength variables, ankle dorsiflexion range of motion, and performance data in training and games in professional Brazilian soccer athletes. Furthermore, an analysis of the external load (GPS) was performed in this study and some authors also recommend the analysis of internal load. The assessment of both the internal and external load and their association with injury risk in soccer players is warranted (64, 65). The HSR distances found in the present study (i.e., medians of 617–730 m for both groups) are in agreement with the standards found in Brazilian football (66). However, the HSR performed by players in this study were lower than previously described in the English Premier League (*i.e.*, ∼778 m) (66). As such, our results may not be generalizable to all competitions around the world. In contrast to our findings, age, eccentric hamstring strength, previous injury history, and concentric hamstring strength at 240° /sec resulted in the best prediction of hamstring muscle injuries in male soccer players of the Kosovo National Premier League (30). Furthermore, the role of eccentric hamstring strength has been studied in soccer context (67) as well as the relationships between exercises used in the training and hamstring fatigue (68–71), thus contributing to the advancement of knowledge on the hamstring risk injury.

To the best of our knowledge, there are still few studies in the scientific literature that have analyzed the costs related to hamstring injuries. In amateur Spanish soccer players, the mean cost of hamstring muscle injuries per player who used the FIFA 11 Programme as their dynamic warm-up was 42% lower: (EUR 742: 95%CI = 410–1,074) compared with athletes who did not use the same approach (EUR 1,271: 95%CI = 929–1,613) (18). In Australian rules football (i.e., Australian Football League), the financial costs associated with recovering from a hamstring injury increased 71% compared with a 43% increase in average yearly athlete salary. From 2003 to 2012, the average financial cost of a single hamstring injury increased by 56%, despite little change in the hamstring injury rates during this period (72). Both cited studies reinforce the findings of an analysis of the financial cost of injuries (not only hamstring injuries), to all ten teams in Australian professional soccer (i.e., A-League) over six consecutive seasons. These researchers found that injury prevention remains necessary for reducing injury-induced athlete-salary costs (73). Allocation of resources for Research, Development, and Innovation is encouraged in the scientific literature aiming to reduce these financial costs and/or increase the earnings on merit money (74–78).

## 5 Limitations

This is a small cohort pilot study, which limits its external validity and the ability to draw firm conclusions. In our mirroring approach, confounders such as age, the number of matches played during the season, eccentric hamstring strength, and previous injury history were present. These cannot be completely controlled in elite soccer players and as such, this represents a limitation of the present study. Furthermore, we analyzed one club during one season; owing to the small sample size it was not possible to perform non-linear and interaction analyses. However, similar approaches can be found in the scientific literature (79, 80).

## 6 Conclusion

The results of this pilot study demonstrate the high burden associated with hamstring injuries in elite soccer players. Hamstring injuries in Brazilian soccer players were associated with a previous hamstring injury, increased fatigue index of knee-flexors, restricted ankle dorsiflexion range of motion, and increased isokinetic hamstring strength asymmetry. In addition, a high potential financial loss was found as a reduced receipt of merit money. Furthermore, our findings provide a path for future studies to analyze the interaction between hamstring muscle injury risk factors and financial burden in professional soccer. Last but not least, this pilot study does not intend to assume that the variables analyzed here are the only ones considered as risk factors for hamstring injuries in soccer.

Practical Applications

1. Significant cost reductions for teams may be achieved with less time spent in rehabilitation because of hamstring injury, and greater availability of athletes for training and games.
2. Allocation of resources for Research, Development, and Innovation (RD&I) Departments to reduce these financial costs and/or increase the earnings on merit money is recommended in soccer clubs.
3. Preventive approaches in professional soccer players with previous hamstring injuries should be a priority.
4. Improvement of ankle mobility, correction of knee-flexor strength deficits and specific training to improve knee-flexor fatigue may reduce hamstring strain injuries.

## Data Availability

The original contributions presented in the study are included in the article/Supplementary Material, further inquiries can be directed to the corresponding author.

## Footnotes

^1^Risk factors such as strength, balance, joint mobility and biomechanics are often of interest as these are modifiable, whereas risk factors such as age and previous injury are non-modifiable. (For more details see Fulton et al. Injury risk is altered by previous injury: a systematic review of the literature and presentation of causative neuromuscular factors. *Int J Sports Phys Ther*. 2014;9(5):583-95).
